# Severe Thrombocytopenia Secondary to Babesiosis: A Case Report

**DOI:** 10.1155/crh/9918329

**Published:** 2025-07-01

**Authors:** Vidyasagar R. Cirra, Sharath Kommu, Michael Husak, Christopher Osterbauer

**Affiliations:** ^1^Department of Hospital Medicine, Marshfield Clinic, Rice Lake, Wisconsin, USA; ^2^Department of Hematology and Oncology, Marshfield Clinic, Rice Lake, Wisconsin, USA

## Abstract

A 76-year-old man with a history of atrial fibrillation treated with warfarin, renal calculi with a history of lithotripsy, hypertension, anxiety, and diabetes mellitus with recent tick exposure presented with abdominal pain, fatigue, nausea, and fever with chills. Workup revealed thrombocytopenia and hemolysis. Due to the likelihood of immune thrombocytopenia (ITP) secondary to a viral etiology, the patient was initially started on steroids. The patient subsequently tested positive for babesiosis on peripheral smear and polymerase chain reaction. A peripheral smear showed giant platelets and was positive for immunoglobulin M platelet antibodies. Other etiologies of thrombocytopenia were excluded. The patient was diagnosed with ITP secondary to babesiosis. Antibiotics were initiated to treat babesiosis. The platelet count was nonresponsive to steroids and gradually improved following intravenous immunoglobulin administration and continued antibiotic treatment. This rare case highlights the importance of considering ITP secondary to babesiosis as the etiology of severe thrombocytopenia in babesiosis, as appropriate recognition and early treatment of babesiosis and ITP can prevent serious complications.

## 1. Introduction

Babesiosis is a parasitic infection caused by protozoans of the genus Babesia. In the United States, human cases are primarily attributed to *Babesia microti*, prevalent in the northeastern and upper-midwestern regions of the country. Tick vectors primarily transmit Babesia. Transmission of Babesia can infrequently occur through blood transfusion and even more rarely through organ transplantation or congenital means. The Babesia protozoa invade and subsequently cause the destruction of host red blood cells (RBC) [1–5]. The clinical presentation of babesiosis ranges from asymptomatic to severe, with a few cases resulting in fatality. Common symptoms of babesiosis include chills, sweats, and myalgia, while less common features comprise headache, arthralgia, abdominal pain, vomiting, photophobia, and weight loss [2]. Physical examination findings may include splenomegaly, hepatomegaly, icterus, and pharyngeal erythema. Diagnosis involves examining a thin blood smear to identify Babesia, along with utilizing polymerase chain reaction (PCR) to detect Babesia deoxyribonucleic acid (DNA) [1].

Severe cases of babesiosis typically entail parasitemia exceeding 4%, with potential complications encompassing acute respiratory distress syndrome (ARDS), pulmonary edema, renal failure, and disseminated intravascular coagulation (DIC) [6, 7]. Risk factors contributing to severe babesiosis include individuals aged over 50 years, those with asplenia, malignancies, and immunosuppressive medication usage [8]. Complications arise in nearly 50% of hospitalized patients with babesiosis [2], and they are significantly correlated with hemoglobin levels below 10 g/dL and high-grade parasitemia exceeding 10% [6]. The mortality rate among hospitalized patients ranges from 3% to 9% [6, 7, 9].

Hematological complications of babesiosis encompass anemia, thrombocytopenia, DIC, among others [10]. Thrombocytopenia in babesiosis is typically mild to moderate and is secondary to hypersplenism, direct platelet consumption and injury, as well as bone marrow suppression [11]. Severe thrombocytopenia associated with babesiosis is rare. Immune thrombocytopenia (ITP) should be considered the leading cause of severe thrombocytopenia secondary to babesiosis.

Treatment for ITP is indicated in the presence of bleeding or if the platelet count is < 20,000/mcL. Glucocorticoids are the preferred initial treatment for ITP. For patients who are unresponsive or unable to tolerate steroids and necessitate a rapid rise in platelet count, intravenous immunoglobulin (IVIG) is recommended [12, 13].

## 2. Case Presentation

A 76-year-old man with a medical history of atrial fibrillation treated with warfarin, renal calculi with a history of lithotripsy, hypertension, anxiety, and diabetes mellitus was admitted to the hospital with concerns of abdominal pain, fatigue, and nausea for one week and fever of 102°F with chills for one day. The patient had a history of significant tick exposure 1 week before the onset of his symptoms. On admission, his vital signs were in the normal range, and oxygen saturation was 94% on room air. On physical examination, he was awake, alert and oriented to place, person, and time. Extraocular movements were intact with no nystagmus. There was no rest tremor, no dysmetria, and no intention tremor on finger-nose-finger testing. Ambulation was normal. The oral mucosa was dry and was noted to have mild icterus without pallor. No lymphadenopathy was found. Cardiovascular and respiratory examination was unremarkable. Abdominal examination revealed mild splenomegaly, without any tenderness and bowel sounds heard. No focal deficits were noted on examination of the central nervous system. Examination of the skin revealed bilateral petechiae of the lower extremities, without any evidence of rash.

Upon admission to the hospital, blood tests revealed a platelet count of 30,000/mcL, hemoglobin level of 12.4 gm/dL, white blood cell count (WBC) of 10,000 per microliter, and fibrinogen of 291 mg/dL. Liver function tests showed elevated levels of aspartate aminotransferase (AST, 109 U/L), alanine transaminase (ALT, 59 U/L), and bilirubin (2.7 mg/dL), predominantly of the indirect type. Serum chemistry showed a sodium level of 131 meEq/L and serum creatinine of 1.4 mg/dL (from a baseline of 1.2 mg/dL). Other test results showed elevated lactic dehydrogenase (LDH, 982 U/L), haptoglobin (< 10 mg/dL), and negative direct Coombs test results. The immature reticulocyte fraction was high (53%), and procalcitonin levels were minimally elevated. The patient was tested negative for coronavirus disease 2019 infection. Computed tomography (CT) of the abdomen showed mild splenomegaly with non-obstructive renal calculi. Warfarin was held in view of severe thrombocytopenia, and prednisone 1 mg/kg/day was initiated with a working diagnosis of thrombocytopenia secondary to ITP associated with viral etiology or tick-borne illness, as per hematology recommendations. Platelet count was normal 1 month before the patient's presentation. Sliding scale insulin for the management of diabetes mellitus was initiated.

During the hospital course, further workup was performed for the evaluation of thrombocytopenia. Vitamin B12, folic acid, antinuclear antibodies (ANA), acute hepatitis panel, antiplatelet antibodies (APA), Babesia smear, Lyme serology, and tick-borne panel were ordered. Doxycycline 100 mg was orally initiated on admission for a likely tick-borne illness, as he was exposed to ticks, and laboratory tests showed elevated liver enzymes and thrombocytopenia. IV fluid administration was initiated due to dehydration. Prednisone 1 mg/kg/day were continued in view of severe thrombocytopenia, likely secondary to ITP associated with a viral or tick-borne illness, as per hematology recommendations.

**Figure 1:**
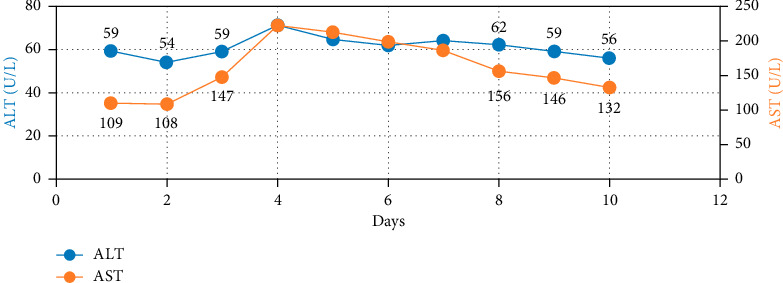
Trend of ALT and AST during hospital course.

**Figure 2:**
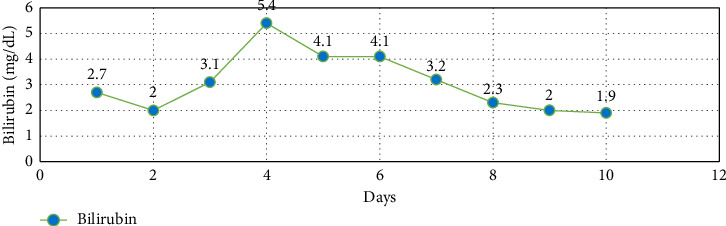
Trend of bilirubin during hospital course.

**Figure 3:**
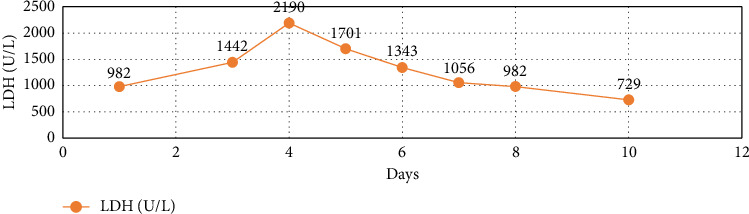
Trend of LDH during hospital course.

**Figure 4:**
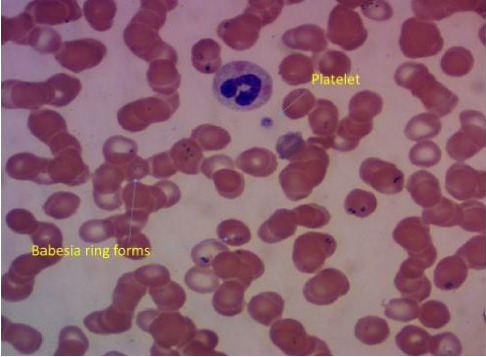
Peripheral blood smear showing Babesia ring forms in red blood cells and sparse large platelet.

Over four days, the platelet count decreased to 17,000/mcL from 30,000/mcL at the time of admission. As noted in Figures 1, 2, 3 and Table 1, Bilirubin, AST, and LDH levels continued to increase during this time, and hemoglobin level was stable at 12.5 g/dL. Lyme serology yielded negative results. B12 and folic acid levels were within normal ranges. ANA is negative. The Babesia smear returned positive after 4 days (Figure 4) with a parasite index of 9.5%. The patient was administered azithromycin (500 mg IV once daily), atovaquone (750 mg orally twice daily), and clindamycin (600 mg IV every six hours) for severe babesiosis following recommendations of an infectious disease physician. Platelet antibodies returned positive for immunoglobulin M (IgM) APA and negative for immunoglobulin G (IgG) antibodies. A peripheral smear revealed thrombocytopenia with predominantly large platelets, as noted in Figure 4. The patient was diagnosed with ITP secondary to babesiosis. Given the continued drop in the platelet count to 9000/mcL over the next two days, IVIG 1 gm/kg was given for two days, and bone marrow aspiration and biopsy were performed. The patient was hemodynamically stable without any episodes of bleeding.

Bone marrow aspiration and biopsy were performed to rule out other causes of thrombocytopenia because of the continued worsening of the platelet count (to < 10,000/mcL). It showed a normocellular marrow with trilineage hematopoiesis and no overt dysplasia. Megakaryocytes were proportional to overall cellularity and did not increase.

**Figure 5:**
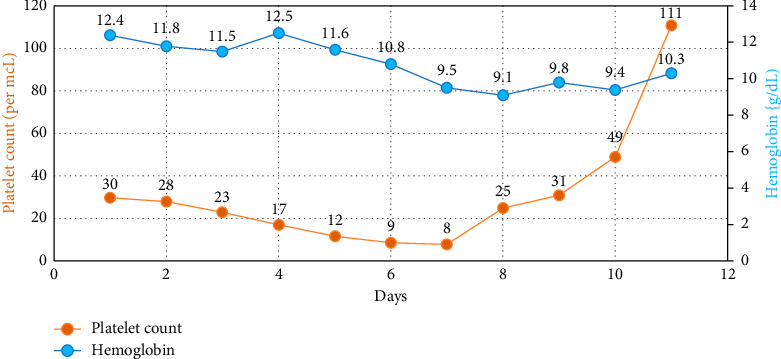
Trend of platelet count and hemoglobin during hospital course.

**Figure 6:**
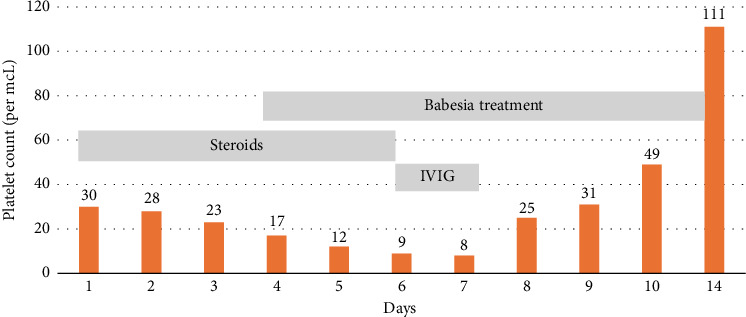
Changes in platelet count during hospital course with various treatments.

**Figure 7:**
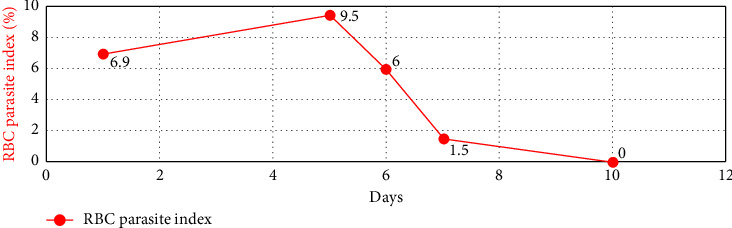
Trend of RBC parasite index during hospital course.

Subsequently, AST, ALT, bilirubin, and LDH levels gradually decreased as noted in Figures 1, 2, 3 and Table 1. Hemoglobin level decreased to 9.1 g/dL at the nadir before improving as noted in Figure 5. The platelet count gradually improved to 49,000/mcL over the next four days, along with significant clinical improvement (Figures 5, 6). The RBC parasite burden improved, and at discharge, no Babesia parasites were noted on the peripheral smear as noted in Figure 7. The patient was discharged on azithromycin and atovaquone to complete babesiosis treatment. On follow-up, four days after discharge, the platelet count increased to 111,000/mcL, and hemoglobin improved to 10.3 gm/dL.

## 3. Discussion

In this case, differential diagnoses included tick-borne illness, acute hepatitis, and sepsis. The patient was empirically initiated on doxycycline for a likely tick-borne illness, given his symptoms and laboratory abnormalities, including transaminitis, thrombocytopenia, and a recent history of tick exposure. Prednisone was initiated for suspected ITP, given the new-onset severe thrombocytopenia without other apparent etiologies of severe thrombocytopenia.

As shown in the case presentation, the initial laboratory abnormalities included hemolysis (elevated bilirubin predominantly indirect type, elevated LDH, decreased haptoglobin, and elevated immature reticulocyte fraction), transaminitis, and severe thrombocytopenia. DIC, thrombotic thrombocytopenic purpura (TPP), acute hepatitis, autoimmune etiology, and sepsis were ruled out as causes of thrombocytopenia.

When the PCR for Babesia and smear for Babesia were positive with a parasite index of 9.5%, severe babesiosis (parasite index > 4%) with hemolysis and ITP secondary to babesiosis was diagnosed, and the patient was initiated on antibiotics for severe babesiosis. When the platelet count dropped to < 10,000/mcL despite being on steroids, he was started on IVIG, given the risk factors for bleeding (age > 60 years, recent use of warfarin), following which his platelet counts improved, and the patient was discharged on oral antibiotics. Hemoglobin trended down to a nadir of 9.4 gm% before improving at discharge, which was considered secondary to hemolysis.

Thrombocytopenia in babesiosis is usually mild to moderate, secondary to hypersplenism, direct platelet consumption and injury, and bone marrow suppression [11]; severe thrombocytopenia is rare. Animal models have shown immune reactions targeting platelets during Babesia infection. Orinda et al. reported ITP induced by autoantibodies against phosphatidyl-serine in cattle infected with Babesia bovis [14], while Lewis et al. identified APA in the serum of dogs affected by babesiosis and ehrlichiosis [15, 16].

ITP is a condition characterized by acquired thrombocytopenia resulting from autoantibodies targeting platelet antigens, leading to platelet destruction and impaired platelet production. Primary ITP, which accounts for 80% of cases, arises from autoimmune mechanisms not associated with other conditions, while secondary ITP (20% of cases) is linked to chronic disorders and infectious diseases. Diagnosing ITP typically involves excluding other possible causes of thrombocytopenia, as there is no definitive test specifically for ITP. However, the pathogenesis of ITP remains unclear. The primary cause of thrombocytopenia is a shortened platelet lifespan due to clearance. This is primarily driven by specific autoantibodies, mainly directed against platelet membrane glycoproteins such as GPIIb/IIIa [17–21]. Additionally, other mechanisms contribute to the pathogenesis, including platelet autoantigen-reactive cytotoxic T cells, dysfunctional T regulatory cells, anomalies in megakaryocyte maturation, and loss of Th1/Th2 balance [22–25].

ITP was primarily suspected in this case, given the severe thrombocytopenia and the exclusion of other etiologies, including DIC, acute hepatitis, sepsis, autoimmune etiology, TTP, and aplastic anemia. Giant platelets on a peripheral smear and positive IgM APA also suggested ITP, although they were not required for diagnosis. Bone marrow aspiration and biopsy were not indicated for ITP diagnosis; however, these were performed in this case to rule out other etiologies of thrombocytopenia, including myelodysplastic syndromes, given his age and continued worsening of thrombocytopenia with the appropriate dose of steroids. Bone marrow can be normocellular or show increased megakaryocytes in patients with ITP. In the present case, the bone marrow showed normal cellularity.

Treatment for ITP is warranted in the presence of bleeding or if the platelet count is less than 20,000/mcL, as most cases of critical or severe bleeding occur at or below this level. In patients with additional risk factors such as age over 60 years, liver or kidney dysfunction, use of antiplatelet agents, anticoagulants, or other medications contributing to bleeding risk, the threshold for initiating treatment is up to 50,000/mcL. Glucocorticoids are the preferred initial treatment for ITP. In patients who are non-responsive to steroids or are intolerant to steroids, where there is a need for a rapid rise in platelet count, IVIG (1 g/kg) as a single dose is administered and repeated the next day unless the platelet count improves to > 50,000/mcL. Management of severe bleeding involves IVIG and IV glucocorticoids. Critical bleeding requires immediate treatment with platelet transfusion along with IVIG and IV glucocorticoids [12, 13].

From a review of the literature, there are only two documented cases of ITP secondary to babesiosis. The first case was reported by Narurkar et al. where ITP (platelet count < 1000/mcL) was most likely triggered by Babesia infection [26]. Parasitemia was only 0.3%, and ITP severity did not correlate with parasitemia. The patient received IVIG, after which the platelet count improved. Another case was reported by Anand et al. where isolated ITP (platelet count of 3000/mcL) was diagnosed secondary to Babesia infection incidentally noted on peripheral smear examination with a parasite load index of only 0.2%, likely due to chronic babesiosis. The patient had partial response to immunosuppressive therapy with steroids and IVIG, and the platelet count improved significantly after the addition of treatment for babesiosis [27].

Pregnancy is an immunocompromised state and risk factor for severe babesiosis with complications, even in patients with a low parasite index. Babesiosis in pregnancy can cause laboratory abnormalities and clinical features which can mimic hemolysis, elevated liver enzymes, and low platelets (HELLP) syndrome. Careful consideration for babesiosis in pregnancy is needed, especially in people who live in or have traveled to endemic areas with symptoms and have laboratory abnormalities like those in HELLP syndrome. With rapid diagnosis, prompt therapy for babesiosis can be instituted to avoid preterm delivery and improve the maternal outcome. Eight cases of babesiosis in pregnancy were documented in a study by Khangura et al. with variable clinical presentations and laboratory features like those of HELLP syndrome [28].

Another case of babesiosis in pregnancy was documented by Pashankar et al. in a 44-year-old woman who was 35 weeks pregnant with dichorionic, diamniotic twins. She presented with fever, weakness, and shortness of breath and had laboratory features mimicking HELLP syndrome. She was found to have normal blood pressure and the peripheral smear diagnosed B. microti infection with 19.3% parasitemia. She was given appropriate therapy for babesiosis and blood transfusion for anemia, along with platelet transfusion. Following emergency caesarean delivery, one of the twins was diagnosed with asymptomatic babesiosis on a peripheral smear collected on day 4 after delivery, suggesting congenital transmission. The twins were treated with oral azithromycin and oral atovaquone with a good outcome. The patient had a complicated course of severe babesiosis, with eventual improvement [29].

Babesiosis can also trigger hemophagocytic lymphohistiocytosis (HLH). HLH is an aggressive and potentially life-threatening syndrome of excessive immune activation. HLH is most common in infants and young children, but it can affect patients of any age group. HLH is associated with an inability to restrict immune responses of activated macrophages and lymphocytes [30]. Many patients, especially infants and children, have an inherited abnormality of perforin-dependent cytotoxicity, while others have an underlying immunologic trigger, such as infection (bacterial, viral or fungal), malignancy, or rheumatologic disorder. Most patients are acutely ill with multisystem involvement but can have varied presentations. Most common signs, symptoms and laboratory findings include fever, rash, hepatosplenomegaly, lymphadenopathy, rash, neurologic symptoms, cytopenias, liver function test abnormalities, and high serum ferritin.

Immune response to a severe babesiosis shares many characteristics with a dysregulated hyperinflammatory response such as seen in HLH. HLH is dominated by a CD8 + response with significant increases in cytokines including IFN-y, resulting in pathologic T-cell activation consistent with a dysregulated immune response [30]. HLH secondary to babesiosis can occur with any parasitemia levels. Most patients are immunocompromised at baseline due to an underlying disease or medication induced immunosuppression.

HLH is difficult to diagnose and needs a high index of suspicion. HLH-2024 criteria include fever, splenomegaly, bicytopenia, hypofibrinogenemia, hyperferritinemia, hemophagocytosis, elevated soluble CD25. A minimum of 5 criteria should be met for the diagnosis of HLH [31]. Many reported patients with HLH due to babesiosis recovered with treatment of the underlying infection without need of immunomodulator therapy.

The patient in this case is noted to have only a few criteria of HLH, and the H-score for this patient was only 99 points, which is below the 169-point cutoff where HLH is highly suspected. H-score is calculated from immunosuppression status, cytopenias, temperature, organomegaly, serum ferritin, triglycerides, ferritin, and AST [32]. Additionally, bone marrow aspirate did not reveal any evidence of hemophagocytosis.

Some of the cases of babesiosis with thrombocytopenia are included in Table 2.

In our case, thrombocytopenia severity correlated well with parasitemia, and platelet count improved following the improvement in parasitemia. Platelet count did not respond to steroids in this case, likely secondary to the delay in initiating antibiotics because babesiosis was diagnosed relatively late. The patient's platelet count improved following IVIG and antibiotics for babesiosis. Hemoglobin trended down during the hospital course, which was likely secondary to hemolysis, and trended up at the time of discharge following improvement in hemolysis. Laboratory features of hemolysis (LDH, bilirubin, and liver enzymes) improved at discharge as noted in Figures 1, 2, 3. The fact that platelet antibodies were IgM suggests that Babesia infection is the causative factor of ITP.

## 4. Conclusions

Severe thrombocytopenia associated with babesiosis is rare, and ITP should be considered the likely underlying cause. The exact pathogenesis of the immune reaction has not been elucidated; however, it appears to be mediated by the host response to the infecting Babesia parasite. The patient's previously normal platelet count and the fact that the platelet antibodies were of the IgM type suggested a primary immune response. Careful consideration for ITP in cases of severe thrombocytopenia associated with babesiosis is required, especially in people who have traveled to or resided in endemic areas, for early diagnosis and effective treatment.

## Figures and Tables

**Table 1:** Labs during hospital course.

Days	Platelet count	Hemoglobin	ALT	AST	LDH	Bilirubin	RBC parasite index
1	30	12.4	59	109	982	2.7	6.9
2	28	11.8	54	108		2.0	
3	23	11.5	59	147	1442	3.1	
4	17	12.5	71	221	2190	5.4	
5	12	11.6	65	212	1701	4.1	9.5
6	9	10.8	62	198	1343	4.1	6.0
7	8	9.5	64	186	1076	3.2	1.5
8	25	9.1	62	156	982	2.3	
9	31	9.8	59	146		2.0	
10	49	9.4	56	132	729	1.9	0
14	111	10.3					

*Note:* Hemoglobin: 12.9–17.3 g/dL. Platelet count: 150–450 per mcL. AST: 15–46 U/L. ALT: 12–70 U/L. Bilirubin: 0.4–1.4 mg/dL. LDH: 85–227 U/L.

Abbreviations: ALT: alanine transaminase; AST: aspartate aminotransferase; IVIG: intravenous immunoglobulin; LDH: lactate dehydrogenase; RBC: red blood cell.

**Table 2:** Cases of babesiosis with clinical features, laboratory findings and treatment.

Case	Clinical features	Laboratory findings	Treatment
Case of isolated immune thrombocytopenic purpura (ITP) in a woman secondary to Babesiosis [26].	Flu-like symptoms, excessive bruises, splenomegaly, diffuse petechial rash, and ecchymosis.	Platelet count of < 1000/mcL. Babesia parasite load of 0.5%. Platelet antibody positive.	IVIG, antibiotics for Babesiosis.
Case of 55 year-old woman with isolated immune thrombocytopenic purpura (ITP) secondary to Babesiosis [27].	Acute onset of petechiae and ecchymosis of the extremities.	Platelet count of 3000/mcL. Babesia parasite load of 0.2%.	IVIG, dexamethasone, azithromycin and atovaquone.
44 year-old woman, with 35 weeks gestation with dichorionic, diamniotic twins with pancytopenia secondary to Babesiosis [29].	Myalgia, fever, shortness of breath.	Platelet count of 8000/mcL. Hemoglobin: 10.7 g/dL. White blood cell count of 1.8 × 10^9^. Parasite load of 19.3%. AST: 110 U/L. ALT: 32 U/L. LDH: 1378 U/L.	Clindamycin, quinine, plasma exchange, platelet transfusion, blood transfusion and emergent delivery of fetus.
70 year-old female with pancytopenia secondary to Babesiosis [33].	Fever, myalgia, sweats, and fatigability	Platelet count of 45,000/mcL. Parasite load of 4%. Hemoglobin of 6.8 g/dL. White blood cell count of 1.8 × 10^9^.	Atovaquone and clindamycin
31-year-old woman, with 37 weeks gestation with thrombocytopenia secondary to Babesiosis.	Headache, abdominal pain and nausea.	Platelet count of 79,000/mcL. AST: 61 U/L. ALT: 59 U/L. Parasite load: 0.71%.	Clindamycin and quinine. Delivery at term.
74 year-old woman with anemia, thrombocytopenia and lactic acidosis from hemophagocytic lymphohistiocytosis triggered by Babesiosis [34].	Fatigue, myalgia and non-bloody diarrhea. Splenomegaly on examination.	Hemoglobin 5.6 g/L. Platelet count of 65,000/mcL. Parasite load: 5%. Lactic acid: 27 mmol/L. Ferritin: 22,156 ng/mL. CD25: 18,068 U/mL. CXCL9: 210,476 pg/mL	Fatal outcome. Died on comfort measures.

## Data Availability

The data used for this case report is included in Table 1 of this article.

## References

[1] Krause P. J., Auwaerter P. G., Bannuru R. R. (2021). Clinical Practice Guidelines by the Infectious Diseases Society of America (IDSA): 2020 Guideline on Diagnosis and Management of Babesiosis. *Clinical Infectious Diseases*.

[2] Vannier E., Krause P. J. (2012). Human Babesiosis. *New England Journal of Medicine*.

[3] Krause P. J. (2019). Human Babesiosis. *International Journal for Parasitology*.

[4] Sanchez E., Vannier E., Wormser G. P., Hu L. T. (2016). Diagnosis, Treatment, and Prevention of Lyme Disease, Human Granulocytic Anaplasmosis, and Babesiosis: A Review. *JAMA*.

[5] Vannier E. G., Diuk-Wasser M. A., Ben Mamoun C., Krause P. J. (2015). Babesiosis. *Infectious Disease Clinics of North America*.

[6] Hatcher J. C., Greenberg P. D., Antique J., Jimenez-Lucho V. E. (2001). Severe Babesiosis in Long Island: Review of 34 Cases and Their Complications. *Clinical Infectious Diseases*.

[7] White D. J., Talarico J., Chang H. G., Birkhead G. S., Heimberger T., Morse D. L. (1998). Human Babesiosis in New York State: Review of 139 Hospitalized Cases and Analysis of Prognostic Factors. *Archives of Internal Medicine*.

[8] Mareedu N., Schotthoefer A. M., Tompkins J., Hall M. C., Fritsche T. R., Frost H. M. (2017). Risk Factors for Severe Infection, Hospitalization, and Prolonged Antimicrobial Therapy in Patients With Babesiosis. *The American Journal of Tropical Medicine and Hygiene*.

[9] Krause P. J., Gewurz B. E., Hill D. (2008). Persistent and Relapsing Babesiosis in Immunocompromised Patients. *Clinical Infectious Diseases*.

[10] Woolley A. E., Montgomery M. W., Savage W. J. (2017). Post-Babesiosis Warm Autoimmune Hemolytic Anemia. *New England Journal of Medicine*.

[11] Pantanowitz L. (2003). Mechanisms of Thrombocytopenia in Tick-Borne Diseases. *The Internet Journal of Infectious Diseases*.

[12] Provan D., Arnold D. M., Bussel J. B. (2019). Updated International Consensus Report on the Investigation and Management of Primary Immune Thrombocytopenia. *Blood Advances*.

[13] Neunert C., Terrell D. R., Arnold D. M. (2019). American Society of Hematology 2019 Guidelines for Immune Thrombocytopenia. *Blood Advances*.

[14] Orinda G. O., Commins M. A., Waltisbuhl D. J., Goodger B. V., Wright I. G. (1994). A Study of Autoantibodies to Phosphatidyl-Serine in Babesia Bovis and Babesia Bigemina Infections in Cattle. *Veterinary Immunology and Immunopathology*.

[15] Lewis D. C., McVey D. S., Shuman W. S., Muller W. B. (1995). Development and Characterization of a Flow Cytometric Assay for Detection of Platelet-Bound Immunoglobulin G in Dogs. *American Journal of Veterinary Research*.

[16] Lewis D. C., Meyers K. M., Callan M. B., Bücheler J., Giger U. (1995). Detection of Platelet-Bound and Serum Platelet-Bindable Antibodies for Diagnosis of Idiopathic Thrombocytopenic Purpura in Dogs. *Journal of the American Veterinary Medical Association*.

[17] Cines D. B., Blanchette V. S. (2002). Immune Thrombocytopenic Purpura. *New England Journal of Medicine*.

[18] Michel M., Lee K., Piette J. C. (2002). Platelet Autoantibodies and Lupus-Associated Thrombocytopenia. *British Journal of Haematology*.

[19] Kuwana M., Kaburaki J., Okazaki Y., Miyazaki H., Ikeda Y. (2006). Two Types of Autoantibody-Mediated Thrombocytopenia in Patients With Systemic Lupus Erythematosus. *Rheumatology*.

[20] Nugent D., McMillan R., Nichol J. L., Slichter S. J. (2009). Pathogenesis of Chronic Immune Thrombocytopenia: Increased Platelet Destruction and/or Decreased Platelet Production. *British Journal of Haematology*.

[21] Toltl L. J., Arnold D. M. (2011). Pathophysiology and Management of Chronic Immune Thrombocytopenia: Focusing on What Matters. *British Journal of Haematology*.

[22] Ji X., Zhang L., Peng J., Hou M. (2014). T Cell Immune Abnormalities in Immune Thrombocytopenia. *Journal of Hematology & Oncology*.

[23] Rocha A. M., Souza C., Rocha G. A. (2013). The Serum Levels of the Cytokines Involved in the Th17 and Th1 Cell Commitment Are Increased in Individuals With Borderline Thrombocytopenia. *Journal of Hematology & Oncology*.

[24] Yu L., Zhang C., Zhang L., Shi Y., Ji X. (2015). Biomarkers for Immune Thrombocytopenia. *Biomarker Research*.

[25] Zhang X. H., Wang Q. M., Zhang J. M. (2015). Desialylation Is Associated With Apoptosis and Phagocytosis of Platelets in Patients With Prolonged Isolated Thrombocytopenia After Allo-HSCT. *Journal of Hematology & Oncology*.

[26] Narurkar R., Mamorska-Dyga A., Agarwal A., Nelson J. C., Liu D. (2017). Babesiosis-Associated Immune Thrombocytopenia. *Stem Cell Investigation*.

[27] Anand L., Vojnic M., Spaccavento C. (2019). Serendipitous Finding of Asymptomatic Babesiosis in a Patient With Symptomatic Thrombocytopenia. *J Hematol*.

[28] Khangura R. K., Williams N., Cooper S., Prabulos A. M. (2019). Babesiosis in Pregnancy: An Imitator of HELLP Syndrome. *American Journal of Perinatology Reports*.

[29] Pashankar R., Prabulos A. M., Feder H. M. (2022). A Woman Pregnant With Twins Has Fever, Haemolysis, and Thrombocytopenia Caused by Babesiosis: Could Be Confused With HELLP Syndrome. *The Lancet*.

[30] Filipovich A., McClain K., Grom A. (2010). Histiocytic Disorders: Recent Insights Into Pathophysiology and Practical Guidelines. *Biology of Blood and Marrow Transplantation*.

[31] Henter J. I. (2025). Hemophagocytic Lymphohistiocytosis. *New England Journal of Medicine*.

[32] Fardet L., Galicier L., Lambotte O. (2014). Development and Validation of the HScore, A Score for the Diagnosis of Reactive Hemophagocytic Syndrome. *Arthritis & Rheumatology*.

[33] Akel T., Mobarakai N. (2017). Hematologic Manifestations of Babesiosis. *Annals of Clinical Microbiology and Antimicrobials*.

[34] Jacobs M. W., Rocco J. M., Andersen L. K., Robertson T. E. (2025). Babesiosis With Low Parasitemia as a Cause of Secondary Hemophagocytic Lymphohistiocytosis in a Previously Healthy Adult. *IDCases*.

